# Knowledge transfer: a worldwide challenge in child mental health: a recommendation to the readership of CAPMH concerning the revised version of the IACAPAP Textbook of Child and Adolescent Mental Health

**DOI:** 10.1186/s13034-018-0220-9

**Published:** 2018-02-17

**Authors:** Anna T. Maier, Rebecca C. Brown, Joerg M. Fegert

**Affiliations:** Department of Child and Adolescent Psychiatry/Psychotherapy, University of Ulm, Steinhoevelstr. 5, 89075 Ulm, Germany

**Keywords:** Advanced education, Knowledge transfer, Knowledge dissemination, Open access

## Abstract

**Background:**

Transfer of knowledge is an important issue throughout all scientific disciplines, especially in the medical and psycho-social field. The issue of worldwide knowledge transfer in child mental health is one of the aims and goals of the journal *Child and Adolescent Psychiatry and Mental Health* (CAPMH). The demand for mental health training is high worldwide, and especially in low- to lower-middle income countries, where inadequate access to knowledge resources in the field of child and adolescent mental health (CAMH) is prevalent. At the same time, many of these countries are showing an increased risk for mental health issues in children and adolescents. The International Association for Child and Adolescent Psychiatry and Allied Professions (IACAPAP) *Textbook of Child and Adolescent Mental Health* counters this problem. It is an open-access e-textbook aiming to provide an overview of current and established treatment and practical approaches for child and adolescent psychiatrists, psychotherapists and allied (mental health) professionals worldwide. First published in 2012, the updated and revised version was launched in 2015. The aim of this commentary is to review and disseminate the usefulness and practicability of content and further material included in the new version of the textbook.

**Review:**

Overall, the textbook contains ten sections divided into 59 chapters, with a total of 1435 pages. The original version of the textbook was written in English. The revised version contains translations of 49 chapters into different languages (to date French, Spanish, Hebrew, Arabic, Portuguese, Russian, Norwegian and/or Japanese), with additional material for knowledge dissemination and self-directed learning (e.g. videos and quizzes) for several chapters. The textbook and the add-on materials for dissemination are of high quality and convey a great introduction to important topics concerning mental health. Apart from knowledge transfer, there is a pragmatic focus on clinical practice and on regional and cultural differences.

**Conclusion:**

The textbook is a new and unique opportunity for professionals all over the world to improve their knowledge, skills and expertise in CAMH. High-quality, up-to-date and freely accessible materials in the field of CAMH are combined with the opportunity to share insights with colleagues.

## Background

Transfer of knowledge is an important issue throughout all scientific disciplines. The “transmission gap” between current scientific knowledge and clinical practice can be especially problematic in the field of medicine and psycho-social studies. The German Council of Science and Humanities strongly suggests for knowledge transfer to be a fundamental part of every educational facility’s strategy. Therefore, the third mission of universities, besides research and teaching, should be transfer and dissemination of knowledge beyond lecture halls [1].

The International Association for Child and Adolescent Psychiatry and Allied Professions (IACAPAP) is a nongovernmental organization with the objectives to support organizations committed to improving mental health of children and adolescents, disseminating information, fostering training, and strengthening bonds between different regions in regard to child and adolescent mental health (CAMH) [2]. Knowledge transfer in CAMH is one of the core aims of the journal Child and Adolescent Psychiatry and Mental Health (CAPMH). Together with other dissemination activities, the journal tries to contribute to free open access communication of research results in the field of CAMH all over the world. In 2012, Joseph M. Rey succeeded in bringing together a worldwide team of knowledgeable experts in CAMH to publish the first version of the IACAPAP Textbook of Child and Adolescent Mental Health. In large parts of the world (i.e. Africa, Eastern Mediterranean, Southeast Asia, and Western Pacific), the gap between available CAMH professionals and population numbers is enormous [3, 4] and training of CAMH professionals, or of generalists in CAMH skills, is often inadequate [3, 5–7]. To compound matters, many children and adolescents live at an increased risk for mental disorders in those countries [5, 8]. Because of the identified need and extremely limited resources for CAMH, an affordable but useful solution was called for [6, 9]. In this regard, textbooks are highly convenient for transfer of knowledge and standards. By offering an electronic textbook, costs are minimized and availability in all regions of the world is maximized [3, 7, 10].

The IACAPAP textbook addresses different objectives of IACAPAP at once: it offers broad high-quality knowledge of CAMH, is available online all over the world and consists of chapters written by international expert authors from different CAMH professions. The target audience for the textbook are professionals training or practicing in CAMH and allied professions, such as general health professionals or social workers.

In some other medical disciplines, open-access textbooks have been published for similar reasons, for example *“A Textbook of Gynecology for Less*-*Resourced Locations”* or the *“Oxford Textbook of Medicine”*, which are available via the WHO for low- and low-middle-income countries [11, 12]. To our knowledge, no independent reviews of these books have been published so far.

After the first version of the IACAPAP textbook had been published, the editors mentioned that there was still room for improvement, for example the need to overcome language barriers. Readers were asked to provide feedback on their experiences with the textbook. In 2015, an updated and revised version of the textbook was published. The new version offers additional and updated chapters, more than three-quarters of the chapters have been translated into different languages, and additional tools for dissemination and ongoing learning have been included [13].

The editor Joseph M. Rey describes the 2015 edition as a tool “to teach child and adolescent mental health from Addis Ababa to Vilnius and from the Universidade federal do Tocantins in Brazil to Yale University in the US.” [13, p. 14]. The textbook aims to provide an overview of, and an introduction to, current and established treatments and practices for professionals worldwide [13].

The textbook is available as an open-access e-book on the IACAPAP website and can be downloaded chapter by chapter (http://iacapap.org/iacapap-textbook-of-child-and-adolescent-mental-health).

The aim of this manuscript is to review and summarize the great usefulness and practicability of content and further materials included in the new version of the *IACAPAP Textbook of Child and Adolescent Mental Health*.

## Review of the IACAPAP Textbook

### Structure, content and availability

The textbook contains 10 sections divided into 59 chapters, comprising 1435 pages.

An introductory section provides an overview of general topics concerning CAMH and ends with a section addressing different topics related to the field, for example emergencies, alternative treatments, clinical quality and patient safety, the United Nations’ Convention on the rights of the child and historical aspects.

Seven of the remaining eight sections approach specific disorders: “*Perinatal and early childhood risk and protective factors”,* which includes early maltreatment and failure in primary health care (associate editor: L. Newmann); *“Developmental disorders”,* for example autism spectrum disorders, underachievement and learning difficulties, enuresis and encopresis (associate editor: J. Liu); *“Externalizing disorders”*, for example attention deficit hyperactivity disorder and its non-pharmacologic treatment (associate editor: F. D. Zepf); *“Mood disorders”,* for example depression, suicide and self-harming behaviors (associate editor: G. Walter); *“Anxiety disorders”,* for example separation anxiety, obsessive compulsive disorder, reactions to trauma *(associate editor: C. Soutullo); “Substance use disorders*”, including alcohol, cannabis and other substance use and misuse; and *“Other disorders”,* including eating-, tic-, borderline personality- and schizophrenic disorders, atypical gender development and problematic internet use. The section *“Psychiatry and Pediatrics”* outlines non-psychiatric conditions in children and adolescents which have a psychiatric background, including somatoform and sleep disorders, epilepsy, HIV/AIDS, or pediatric delirium (associate editor: A. S. Martin).

Since 2013, 18 new chapters were added to the textbook; three in 2013, four new chapters both in 2014 and 2015 and another seven new chapters since 2016 [13].

The chapters provide a thorough introduction to a large variety of topics. For additional information, literature suggestions and links are provided in each chapter. As an example, the chapter *“Early maltreatment and exposure to violence”* (S. M. K. Tan, N. K. Nor, L. S. Fong, S. Wahab, S. Marimuthu and C. L. Fong) gives a good introduction to and a clear overview of the topic. Different types of maltreatment are explained and difficulties in finding a definition of child maltreatment are depicted in a differentiated and understandable manner. An array of graphs, synoptic tables and video-clips illustrate the contents of the chapter. The influence of toxic stress on the development of a child is, for example, explained in a video-clip. Additionally, practical advice is given and strategies for prevention of child maltreatment are outlined. As a minor critical point, whereas sexual abuse is examined in detail, a more thorough presentation of the other kinds of child maltreatment would be desirable.

Additionally, presentations for dissemination of the contents are available in the new version on 17 topics.

Self-assessment exercises for self-directed learning are available for several chapters of the new version. In our opinion, it would be worthwhile to offer both, prepared presentations and self-assessment exercises, in all chapters.

The original version of the textbook is in English, but with the new version, 49 chapters are available in French, Spanish, Hebrew, Arabic, Portuguese, Russian, Norwegian and/or Japanese. The range of available translations are constantly being extended [13].

The textbook can be downloaded chapter by chapter in separate PDF files, which ensures availability even in regions and facilities with restricted internet access. The presentations are offered as Microsoft PowerPoint files, which can also be opened with older and free presentation software. In some chapters, hyperlinks to videos are used. Most of the videos are available on YouTube and therefore accessible in most parts of the world. Other hyperlinks to reports, further information or important homepages are also directly accessible in the PDF files. Therefore, while reading the chapters as an introduction, hyperlinks can be used for obtaining more detailed and in-depth information.

In addition, there is also an extremely useful app available for smartphones and tablets, which can be downloaded for free and is optimized for offline reading (“IACAPAP Text” available for iOS and Android). Users of the app can access all contents of the textbook and be notified of updates.

### Reader-friendliness

The different chapters are coherent and easy to read, texts are structured consistently and clearly. The frequent use of illustrations adds positively to reading comprehension. Each chapter provides keynotes of the topic, highlighted in special boxes. An example is the chapter *“The clinical examination of children, adolescents and their families*” (T. Lempp, D. de Lange, D. Radeloff and C. Bachmann). It starts with an exceedingly helpful guide ‘Ways to read this chapter’, that supports the different target groups in getting familiar with any sections which are especially interesting to them. Furthermore, the authors point out the importance and relevance of the different topics covered before approaching aims, strategies, methods, and tools concerning the examination. The chapter is clearly and logically structured and completed by practical advice in textboxes, charts and videos. It ends with advice on communication and on how to proceed in cases of unclear diagnosis.

Following this example of great achievement in order to further improve reading comprehension, at the beginning of each chapter a summary and a guide on how to read the chapter would be useful throughout the textbook.

In conclusion, it is advisable to read the textbook in the electronical version, as the printed version does not support several tools such as hyperlinks or the detailed presentation of complex figures. Thanks to the modular character of the textbook, the single chapters can be updated continuously.

### Practical relevance

Apart from knowledge transfer, many of the authors focus on the everyday work of CAMH and allied professionals (e.g. also general health professionals such as nurses or general practitioners and social workers) and offer some highly important indications and tools for professional practice (e.g. questionnaires for psychological assessment). The frequent use of practical examples raises the practical usefulness of the contents of each chapter. Simple advice and practical points to consider, along with the extensive use of illustrations, are particularly useful for busy practitioners. Furthermore, in several chapters, numerous case studies are provided, helping to enhance internalization of the topic. In some of the chapters, questions concerning these case studies are available, which further promotes transfer of learned contents into daily practice.

For example, in the chapter on selective mutism (B. Oerbeck, K. Manassis, K. Romvig Overgaard, and H. Kristensen), several case studies are provided after a theoretical introduction and description of the topic. These are meant to illustrate the range of presentations and promote the examination of different treatment options for selective mutism. One of the case studies has to be solved autonomously by the readers.

The authors of some chapters encourage to submit comments and even offer to answer questions via Facebook, a hyperlink to the website is included in the text [13].

### Knowledge and dissemination

Open access publishing and new digital media provide an unparalleled opportunity of knowledge transfer and dissemination, which is utilized by the IACAPAP textbook. To date, several chapters have provided different materials, such as presentations and case studies, which can be used to disseminate the content of the textbook to other healthcare workers. While some materials and tools are directly included in the chapters, prepared digital presentations are provided separately in each chapter.

The materials provided in the chapters often include videos, which either give an overview or offer deeper insight into the specific topics. In addition, diagnostic instruments are presented and made available, which are also meant to be shared with colleagues. In the chapter *“Autism spectrum disorder”* (J. Fuentes, M. Bakare, K. Munir, P. Aguayo, N. Gaddour, and Ö. Öner) a simple form of dissemination is suggested: The reader is asked to write a summary based on the contents of the chapter and to send it to colleagues. The chapter itself is well structured and informative, and the summary is a possibility to disseminate the most important messages to a broader audience including non-specialists or community health workers. The case studies mentioned above can also be used for group learning by discussing them with colleagues.

The structure of most of the presentations is well thought through and presents the contents of the chapters clearly. In order to enhance readability and to avoid sensory overload, prepared handouts with respective figures or charts can be distributed at presentations. The presentation on “*Attention deficit hyperactivity disorder (ADHD)”* (created by J. Chilton) is, for example, an extremely well-structured and reader-friendly presentation. The learning objectives and importance of the topic is emphasized at the beginning. In the presentation, a stringent procedure addresses epidemiology, course, symptoms, consequences and comorbidities of the disease, etiology, risk factors, diagnostic tools, different treatments and finally hyperlinks for further information. What should be especially highlighted are case studies, which show ADHD in different cultural backgrounds and make it a subject of discussion. The presentation on *“The clinical examination of children, adolescents and their families”* (created by H. Klasen) combines theoretical input and practical methods on how to deal with patients and their families. Additionally, different exercises are included in the presentation in order to promote application of the learned contents.

A particular welcome feature is that all presentations can be edited by the reader, which is a huge advantage for preparing individual presentations for dissemination.

## Conclusion

Joseph M. Rey has created a unique possibility for CAMH and allied professionals (e.g. healthcare and social workers) to improve their expertise, by learning about best practice and sharing their insights with colleagues. In summary, both texts and add-on materials for dissemination are of high quality, easily accessible, and free of charge.

The textbook stands out from established e-books and textbooks, first of all because of the free availability combined with the high quality of content. The modular nature, which allows downloading chapter by chapter, easy addition of new chapters, and continuous updating of the contents as well as the implementation of readers’ suggestions, is outstanding. The contents of the textbook are comprehensive and have practical relevance.

The collaboration of authors from countries and cultures all over the world will ensure international relevance which supports global dissemination and worldwide usage of the textbook. The various efforts of the authors and editors to overcome regional disparities and to make the textbook more attractive for a broader audience by customizing it to the different regions should be retained.

Last but not least, it is noteworthy that the IACAPAP textbook has created an outstanding opportunity to reach as many professionals in the field of CAMH and allied professions as possible, by offering an easy-access way to acquire high-quality information. There is no doubt that the textbook by the IACAPAP and Joseph M. Rey meets its intended purpose.

## Abbreviations

ADHD: attention deficit hyperactivity disorder
CAMH: child and adolescent mental health
CAPMH: Child and Adolescent Psychiatry and Mental Health
IACAPAP: International Association for Child and Adolescent Psychiatry and Allied Professions

## References

[1] Fegert JM, Brown R, Harsch D, Rassenhofer M, Hoffmann U. Wissenstransfer, Dissemination, E-Learning: 10 Jahre webbasierter Wissenstransfer an der Klinik für Kinder- und Jugendpsychiatrie/Psychotherapie Ulm. Ulm: Klinik für Kinder- und Jugendpsychiatrie/Psychotherapie Ulm; 2017.

[2] International Association for Child and Adolescent Psychiatry and Allied Professions (IACAPAP): About IACAPAP. 2011. http://iacapap.org/about. Accessed 27 Sept 2017.

[3] Chan R (2013). Book review: IACAPAP Textbook of Child and Adolescent Mental Health. Australasian Psychiatry..

[4] World Health Organization (editor). Atlas: Child and Adolescent Mental Health Resources: Global concerns, implications for the future. Geneva: World Health Organization; 2005.

[5] Robertson B, Omigbodun O, Gaddour N (2010). Child and adolescent psychiatry in Africa: luxury or necessity?. Afr J Psychiatry..

[6] Kieling C, Baker-Henningham H, Belfer M, Conti G, Ertem I, Omigbodun O (2011). Child and adolescent mental health worldwide: evidence for action. Lancet.

[7] Rey J, Omigbodun O (2015). International dissemination of evidence-based practice, open access and the IACAPAP Textbook of Child and Adolescent Mental Health. Child Adolesc Psychiatry Ment Health..

[8] UNICEF. Division of Data, Research and Policy. Generation 2030 Africa. United Nations Children’s Fund. 2014. https://www.unicef.org/publications/files/Generation_2030_Africa.pdf. Accessed 27 Sept 2017.

[9] Omigbodun O, Lowell Belfer M (2016). Building research capacity for child and adolescent mental health in Africa. Child Adolesc Psychiatry Ment Health..

[10] Henderson SW (2013). Electric dreams. J Am Acad Child Adolesc Psychiatry.

[11] Van Beekhuizen H, Unkels R (editors). A textbook of gynecology for less-resourced locations. London: Sapiens Publishing; 2012.

[12] Warrell DA, Cox TM, Firth JD (2010). Oxford textbook of medicine.

[13] Rey JM (2015). IACAPAP e-Textbook of Child and Adolescent Mental Health.

